# Economic values, genetic gains and economic contributions of a selection index for Romosinuano Creole cattle

**DOI:** 10.1007/s11250-026-05293-2

**Published:** 2026-09-08

**Authors:** Juana Moncaleano-Vega, Alejandro Amaya, Carlos Martínez, William Burgos-Paz, Mario Cerón-Muñoz

**Affiliations:** 1 Doctorado en Ciencias Animales, Facultad de Ciencias Agrarias, Universidad de Antioquia, Medellín, Colombia; 2 Facultad de Medicina Veterinaria y Zootecnia, Departamento de Producción Pecuaria. Ibagué, Universidad del Tolima, Tolima, Colombia; 3 Universidad de Ciencias Aplicadas y Ambientales U.D.C.A, Programa de Zootecnia, Bogotá, Colombia; 4 Facultad de Medicina Veterinaria y Zootecnia, Departamento de Producción Animal, Universidad Nacional de Colombia, Bogotá, Colombia; 5 Centro de Investigación Turipaná, Corporación Colombiana de Investigación Agropecuaria-AGROSAVIA, Km 13, vía Montería-Cereté, Córdoba, Colombia; 6 Grupo GAMMA, Facultad de Ciencias Agrarias, Universidad de Antioquia, Medellín, Colombia

**Keywords:** Bioeconomic model, Colombia, Creole cattle, Genetic progress

## Abstract

The objective of this study was to estimate economic values (*ev*), genetic gains (*gg*), and economic contributions (*ec*) of age at first calving (AFC), calving interval (CI), longevity (LONG), weaning weight (WW), weight at 16 months (W16), and weight at 24 months (W24) within a selection index for the Romosinuano breed. Three production scenarios were evaluated according to the selling age of animals (8, 16, and 24 months). Productive and reproductive parameters were obtained from records provided by AGROSAVIA between 1976 and 2021, while economic information was collected using a structured format designed to characterize production systems. The *ev* were calculated as the partial derivative of profit per hectare, and *ec* were derived from *gg* and *ev*. Profit per hectare was 239.7, 198.41, and 294.95 USD in scenarios 1, 2, and 3, respectively. CI showed the most relevant *ev* in scenarios 1 (-0.794 USD) and 3 (-0.979 USD), whereas W16 had the greatest economic value in scenario 2 (1.05 USD). AFC showed negative values across the three scenarios, in contrast to LONG, which presented positive values and considerable *ec* (0.56, 0.34, and 0.77 USD). Furthermore, WW and AFC were important in scenario 1, while W16 and W24 were relevant in scenarios 2 and 3. In conclusion, CI was confirmed as the trait with the greatest economic impact, whereas W16 and AFC were identified as relevant traits to be included in balanced selection indices. Also, the contribution of LONG highlights the role of Romosinuano cattle in resilient and sustainable Colombian beef production systems.

## Introduction

Since the 1940s, selection indices have been developed to optimize genetic response in animal breeding programs (Hazel and Lush 1942). The construction of these indices requires assigning economic values to traits included in a breeding objective. An economic value is trait-specific and defined as the change in production system profit resulting from a unit change in phenotypic performance of that trait. However, this value is not constant, as it depends on the context in which the system operates, including management practices and market conditions.

The linear combination of economic and breeding values estimates the total economic merit of each animal, thereby ranking them for selection. A main challenge in constructing indices is ensuring favorable genetic progress for all traits of interest, particularly when unfavorable correlations exist among them (Mancin et al. 2022). Therefore, appropriate valuation of each trait is essential to maximize profitability through genetic selection (Wolfová et al. 2005; Brumatti et al. 2011; Pravia et al. 2014; Michaličková et al. 2016; Kodak and Nagy 2019; Krupová et al. 2020).

In beef cattle, economic valuation shows that reproduction and adaptation hold greater relative importance than growth traits. This pattern has been reported across production systems, in both tropical and temperate environments, and in purebred as well as crossbred populations (Laske et al. 2012; Koots and Gibson 1998; Albera et al. 2004; Amaya et al. 2022). Earlier maturing and more fertile females increase herd calving rate, reduce culling, and enable greater annual beef output, directly influencing farm costs and revenues (Eler et al. 2014).

Recently, local breeds have gained attention due to their performance in functional traits such as health, fertility, longevity, and resilience compared with cosmopolitan breeds (Mancin et al. 2022). However, for the Colombian Creole breed Romosinuano, no indices have been proposed that integrate these traits. Moreover, no estimates of biological and economic progress based on their application have been reported (Cañas-Álvarez et al. 2023). The objective of this study was to estimate the economic values of relevant traits, and the genetic progress and economic contributions of a proposed index for beef production systems using Romosinuano cattle.

## Materials and methods

***Bioeconomic model***: A deterministic bioeconomic model was developed to estimate profit per hectare and marginal economic values. Three production scenarios were simulated according to the age at which animals were marketed (8, 16, and 24 months). In all scenarios, a cow–calf pasture-based system was assumed, with males destined for beef production, females retained first as replacements, surplus females marketed, and cull cows sold as adult animals. The base herd was standardized to 100 breeding cows, using the productive and reproductive parameters presented in Table 1. A 1:1 sex ratio at birth, fixed survival rates for pre-weaning, post-weaning, growing, and adult stages, and a stable herd size across simulations were assumed.

**Table 1:** Productive parameters of the base system

Parameter	Value
Birth weight (BW)	30 kg
Weaning weight (WW)	162.6 kg
Weight at 16 months (W16)	197.3 kg
Weight at 24 months (W24)	295.9 kg
Age at first calving (AFC)	1187 days
Calving interval (CI)	388 days
Longevity (LONG)	3328 days
Lactation length (LL)	240 days
Milk yield (MY)	960 L

The model considered rotational grazing on *Brachiaria* spp., with fixed forage availability, grazing losses, and net energy concentration. Energy requirements for maintenance, lactation, gestation, and growth were estimated according to NRC (2021) equations and converted into dry matter requirements using the energy concentration of the pasture. Growth trajectories were derived from observed body weights at birth, weaning, 16 months, 24 months, first calving, and adult age.

Economic information was collected using a structured survey designed to characterize herd structure, grazing management, productive performance, sources of income, and production costs. The survey was applied to Romosinuano cattle farmers from three Colombian regions: Costa Atlántica, Urabá Antioqueño, and Norte del Tolima. Production costs and revenues were converted to U.S. dollars, and the reference price per kilogram of live weight was obtained from official Colombian market sources. Income included the sale of males, surplus females, and cull cows, using the live-weight prices corresponding to each scenario, whereas costs included grazing dry matter, mineral supplementation, veterinary, and biosecurity expenses, as summarized in Table 2. Although milk yield was considered to estimate lactation energy requirements, milk sales were not included because the evaluated system was beef.

Profit per hectare was calculated from annual income per cow, non-feed variable costs, forage availability, dry matter intake per cow, and the cost of additional feed. The additional dry matter requirement represented the extra amount of feed required per hectare after the one-unit increase in each trait. It was estimated from the difference between the land area required by the simulated herd after trait perturbation and the land area required by the base herd, expressed in kilograms of dry matter per hectare. This procedure allowed the model to account for the additional feed cost associated with higher body weight, longer productive permanence, or changes in reproductive efficiency.

**Table 2:** Expenses and incomes in the baseline production system

Expenses	
**Feeding**	**USD**
Kilogram of dry matter from grazing	0.040
**Supplementation**	
Kilogram of mineral for breeding females	1.1
Kilogram of mineral for growing animals	0.82
Kilogram of silage	0.262
**Biosecurity**	
Cost per non-pregnant cow	10.20
Cost per calf	11.22
Cost per growing animal	1.6
Cost per breeding female and replacement	10.20
**Incomes**	
**Live weight (kg)**	**USD**
Male, 8 months of age	2.12
Male, 16 months of age	1.72
Male, 24 months of age	2.01
Female, 8 months of age	2.35
Female, 16 months of age	1.96
Female, 24 months of age	1.87
Cull female	1.84

### Economic values, genetic progress, and economic contributions

The first step in estimating economic values was to calculate the baseline profit per hectare (π) for each production scenario. It was defined as the difference between total revenues and production costs, expressed per hectare of grazing land required by the herd. The economic value of a trait ($\:{ev}_{i}$) was then estimated as the change in π resulting from a one-unit increase in the phenotypic value of that trait, while all other biological and economic parameters were held constant, following the methodology proposed by Garrick (2002).

$$\:{ev}_{i}=\frac{\partial\:\pi\:}{\partial\:{z}_{i}}=\left(\frac{F+x}{T}\right)\frac{\partial\:\left(K\right)}{\partial\:{z}_{i}}-C\left(\frac{F+x}{T}\right)\frac{\partial\:T}{\partial\:{z}_{i}}$$

where $\:{z}_{i}$ corresponds to the i-th trait (WW, W16, W24, AFC, CI, or LONG); *F* represents the availability of dry matter (kg/ha) in the production system; *x* denotes the additional kilograms of dry matter required per hectare to meet the extra feed requirements associated with changes in trait performance; *T* is the total annual dry matter intake per cow; *K* refers to income per cow from the sale of weaned calves, growing animals, cull cows, and surplus females; and *C* is the price per kilogram of additional feed. The first term represents the marginal change in revenues, whereas the second term represents the marginal change in feeding costs associated with the trait.

Genetic gains and economic contributions were estimated using the methodology described by Schneeberger et al. (1992). Expected genetic gains were calculated as:

$$\:gg=(1/{S}_{I}^{2}\times\:var({\widehat{u}}_{l})\times\:b)$$

where *gg* is the vector of expected genetic gains for the evaluated traits; $\:{S}_{I}^{2}$ is the variance of the selection index, estimated as $\:{S}_{I}^{2}=b{\prime\:}\times\:var\left({\widehat{u}}_{l}\right)\times\:b$; and $\:var\left({\widehat{u}}_{l}\right)$ approximates the covariance matrix of the predicted breeding values, represented as $\:var\left({\widehat{u}}_{l}\right)={B}^{{\prime\:}}\times\:{P}_{11}\times\:B$, where $\:B={P}_{11}^{-1}\times\:{G}_{11}$. $\:{P}_{11}$ and $\:{G}_{11}$ represent the phenotypic and genetic covariance matrices of the selection criteria, respectively. The corresponding genetic and phenotypic parameter structure is summarized in Table 3. Because a complete covariance matrix for all evaluated traits was not available from a single Romosinuano population, the required phenotypic and genetic parameters were compiled from previous studies in Romosinuano cattle. This approach was adopted to construct a biologically coherent covariance structure for the complete set of traits included in the index, while prioritizing estimates generated under Colombian production conditions (Ossa et al. 2007, 2013, 2021; Galeano 2010; Restrepo 2014; Ramírez et al. 2019, 2020; Gathura et al. 2020). The resulting covariance matrix was therefore considered a biologically informed approximation, given that parameter estimates may vary among studies due to differences in population structure, environmental conditions, trait definitions, and statistical models.

**Table 3:** Genetic and phenotypic parameter structure compiled from the literature for the selection criteria used in the base system

	WW	W16	W24	AFC	CI	LONG
WW	0.13	0.87	0.75	-0.34	0.22	0.1
W16	0.79	0.21	0.94	0.31	0.14	0.18
W24	0.19	0.58	0.26	0.1	0.01	0.35
AFC	0.06	0.24	0.02	0.16	0.21	-0.05
CI	0.2	-0.1	-0.16	-0.3	0.05	0.24
LONG	0.3	0.14	-0.07	0.32	0.1	0.1

Diagonal: heritability. Above the diagonal: genetic correlations. Below the diagonal: phenotypic correlations. WW: weaning weight; W16: weight at 16 months of age; W24: weight at 24 months of age; AFC: age at first calving; CI: calving interval; LONG: longevity

Finally, *b* represents the index weights, corresponding to the economic values estimated from the partial derivatives. The economic contribution (*ec*) for each selection criterion was calculated by multiplying each element of *gg* by its corresponding value in *b*.

## Results

Table 4 presents the profit per hectare per year, income per cow, and feed costs and dry matter intake per cow across the three production scenarios. The highest income per cow was obtained in scenario 3, whereas the lowest was observed in scenario 2. The profit per hectare followed the same trend, with scenario 2 showing a reduction of 41.29 and 96.54 USD compared with scenarios 1 and 3, respectively.

**Table 4:** Profit per hectare of each scenario

Source	Scenario 1	Scenario 2	Scenario 3
Dry matter intake per cow (T, kg DM)	3055.52	3076.49	3254.64
Costs per cow (C, USD)*	109.04	142.15	174.92
Income per cow (K, USD)	370.59	360.16	517.75
Profit per hectare (𝛑, USD)	239.7	198.41	294.95

*Excluding feed costs

Table 5 presents the economic values of the traits. The most economically relevant trait in scenario 1 was CI, with a value of -0.794 USD. In scenario 2, the most important trait was W16 (1.05 USD), while in scenario 3, CI was again the most influential, with − 0.979 USD. Regarding the economic value of LONG, it showed an increase of 0.001 USD between scenarios 2 and 3. In contrast, the increase in AFC did not favor profitability, as its values became more negative as the production system aimed at selling older animals.

**Table 5:** Economic values (USD) of the traits considered by each production scenario

Trait	Unit	Scenario 1	Scenario 2	Scenario 3
WW	Kg	0.576		
W16	Kg		1.050	
W24	Kg			0.206
AFC	d	-0.049	-0.051	-0.053
CI	d	-0.794	-0.690	-0.979
LONG	d	0.021	0.021	0.022

WW: weaning weight; W16: weight at 16 months of age; W24: weight at 24 months of age; AFC: age at first calving; CI: calving interval; LONG: longevity

Genetic gains and economic contributions per unit increase in the index are shown in Table 6. In scenario 1, LONG showed the highest economic contribution, followed by WW (0.56 and 0.25 USD, respectively) and AFC (0.18 USD). In scenario 2, the genetic gains of W16 and LONG were the highest, also generating the greatest economic contributions with 0.66 and 0.34 USD, respectively. In scenario 3, the traits with the greatest impact on the selection index were W24 and LONG, jointly contributing 0.90 USD per dollar of increase in the index. CI did not report either genetic gains or economic contributions in this scenario. Scenario 1 was the only scenario showing favorable responses for all traits, with higher WW and LONG and lower AFC and CI.

**Table 6:** Expected genetic gains in trait units and economic contributions in USD for each trait under different production scenarios

Trait	Scenario 1		Scenario 2		Scenario 3	
	gg	ec	gg	ec	gg	ec
WW (kg)	0.44	0.25				
W16 (kg)			0.63	0.66		
W24 (kg)					0.61	0.13
AFC (d)	-3.63	0.18	-0.12	0.01	-1.98	0.10
CI (d)	-0.01	0.01	0.01	-0.01	0.00	0.00
LONG (d)	26.60	0.56	16.25	0.34	34.90	0.77

WW = weaning weight; W16 = weight at 16 months of age; W24 = weight at 24 months of age; AFC = age at first calving; CI = calving interval; LONG = longevity; *gg* = genetic gain; *ec* = economic contribution

## Discussion

Production systems in tropical regions differ in input levels and productivity (Wahinya et al. 2022). Colombia is no exception, where heterogeneous production objectives and management practices limit the feasibility of a generalized selection index (Cole et al. 2021). Therefore, the evaluation of three production scenarios differing in animal sale age allowed the economic relevance of growth, reproductive, and longevity traits to be interpreted under alternative commercial strategies for Romosinuano cattle.

Although survival rates, reproductive parameters, and forage production were kept constant across scenarios, profit per hectare varied markedly among systems (Table 4). This variation reflects the joint effect of live-weight price, time required to reach market age, feed demand, and herd inventory structure. As animals remain in the system, dry matter intake and non-feed costs increase (Kosińska-Selbi et al. 2022), while revenues depend on whether the additional body weight compensates for the longer production period and the price paid for each animal category (Pérez 2004). Thus, the economic value of a trait should not be interpreted only as a biological response, but as the net result of its effect on revenues and costs within a specific production scenario.

Growth traits had a favorable economic effect across the evaluated scenarios, although their relative importance depended on the age at sale. The greater relevance of W16 suggests that the post-weaning phase represents an efficient biological window in Romosinuano cattle, in which additional body weight can be converted into economic return with comparatively lower maintenance costs. The variability reported among studies for growth-related economic values likely reflects differences in production objectives, input levels, breed type, market prices, and sale endpoints (Schmidtmann et al. 2021; Mezgebe et al. 2018; Amaya et al. 2020, 2022). From a practical breeding perspective, these results support the inclusion of W16 as a relevant selection criterion in Romosinuano systems oriented toward intermediate-age marketing. However, selection for growth should be balanced with reproductive efficiency and longevity, because improvements in body weight may not translate into greater whole-system profitability if they increase feed requirements or generate unfavorable correlated responses in functional traits.

The results of Samaraweera et al. (2022) indicate that prolonged calving intervals negatively affect profit per hectare because of the direct relationship between CI, annual calf output, kilograms of beef sold per production cycle, and revenues derived from calf sales. In the present study, CI also showed a marked negative effect on profitability, confirming that reproductive efficiency is a key determinant of economic performance in beef Romosinuano systems. Biologically, longer CI reduces the number of calves produced per year, increases the proportion of time in which cows consume resources without generating saleable offspring, and reduces beef output per hectare. This explains why CI showed one of the greatest economic impacts among the evaluated traits, particularly in systems where income depends primarily on calf production rather than milk sales.

According to Wahinya et al. (2022), economic values for CI under tropical production conditions range from − 0.89 to − 2.3 USD, with larger effects observed in more intensive systems. In contrast, Mezgebe et al. (2018) reported positive economic values for CI (0.47–0.8 USD), associated with longer lactation length and higher milk revenues relative to calf sales. This situation was not considered in the present study because Romosinuano cattle were evaluated under a beef production system. Therefore, reducing CI represents an important opportunity to improve both biological and economic efficiency. However, the unfavorable response observed for CI in scenario 2 suggests that selection emphasis on post-weaning growth, particularly W16, should not be applied without controlling reproductive traits. In practical breeding programs, W16 may be used as an economically relevant selection criterion, but it should be included in a balanced index with CI, AFC, and LONG to avoid correlated responses that could reduce reproductive efficiency and long-term profitability.

Similarly, all reports in the literature agree that increasing AFC negatively impacts the profit of the system (Hunde et al. 2024), in addition to contributing to a longer generation interval (Kluska et al. 2018). The reduction in profitability associated with higher AFC is mainly driven by increased costs of feed, health, and labor (Samaraweera et al. 2022). Wahinya et al. (2022) reported an economic value under tropical production conditions that is close to that estimated in this study, − 0.036 USD, confirming that higher phenotypic values of AFC increase the production costs of replacement females and negatively affect system profitability. AFC should be considered a functional reproductive trait with direct implications for selection efficiency in tropical beef systems where replacement development is costly and delayed reproductive onset can limit both economic return and genetic progress.

Regarding longevity, the positive economic value was associated with an extended productive life within the herd, and its economic effect has been consistently positive and even more relevant than AFC and CI (Hunde et al. 2024). Ratwan et al. (2021) reported that longevity accounted for 11–14% of the emphasis in selection indices proposed for local breeds, highlighting its relevance in breeding objectives involving adapted genetic resources. The economic impact of improving longevity is mainly explained by changes in herd structure, as a greater proportion of productive cows reduces the need for replacements (Shi et al. 2025). Thus, longevity in beef cattle integrates survival, fertility, adaptation, and cumulative productivity, and its favorable economic effect reflects the ability of cows to remain reproductively functional under tropical production conditions.

However, herd life may lose efficiency when cows remain beyond their most productive years. In this study, peak production was assumed at the third calving and cows remained in the herd until the tenth calving. Previous studies suggest that the optimal length of stay ranges between the fifth and ninth calving, when cows combine greater physiological maturity, higher conception rates, and lower incidence of health and production problems (Cushman et al. 2013; Damiran et al. 2018; Moorey et al. 2020). In Creole cattle, longevity is particularly relevant because adaptation, reproductive resilience, and productive permanence are among the main strengths of these genetic resources. Thus, including LONG in breeding objectives may help preserve adaptive capacity while improving the economic sustainability of Romosinuano production systems.

In terms of genetic gains and economic contributions, the results should be interpreted cautiously until complete multi-trait covariance estimates become available for the Romosinuano population. Nevertheless, the observed responses provide useful information on the expected direction of selection under each production scenario. The only fully favorable outcome was observed in scenario 1, where the index promoted genetic responses consistent with higher profitability. In scenario 3, favorable genetic progress was obtained for AFC, W24, and LONG, while CI showed no genetic progress and made no contribution to economic return. This response can still be considered beneficial for system profitability, because the index improved economically relevant traits without generating an unfavorable correlated response in CI (Mancin et al. 2022).

In contrast, scenario 2 showed an unfavorable economic response associated with a positive genetic gain for CI. This result indicates that, although W16 was economically relevant, selection pressure on post-weaning growth may lead to an undesirable correlated response in reproductive efficiency if the covariance structure among traits is not adequately considered. Such responses are expected in multi-trait selection because genetic correlations are population-specific and may generate antagonism between growth and fertility traits (Zhang and Amer 2021).

More specifically, although the unfavorable response in CI was small in magnitude (0.01), it is biologically and economically informative. In scenario 2, W16 received the largest positive economic value (1.050) and generated the greatest economic contribution (0.66), whereas CI had a negative economic value (− 0.690). However, the expected response of a trait in a multi-trait selection index is determined by the complete covariance structure rather than by its economic value in isolation. The positive, although relatively weak, genetic correlations of CI with W16 (0.14) and LONG (0.24), combined with the positive selection emphasis placed on these traits, likely induced a small indirect increase in CI that was not fully offset by its negative economic value. The low magnitude of these correlations suggests that the genetic antagonism was limited, which is consistent with the small unfavorable response observed for CI.

From a biological perspective, this pattern may reflect a resource-allocation trade-off, whereby genotypes with greater post-weaning growth potential may also have higher feed and maintenance requirements, potentially constraining nutrient allocation to reproductive functions under pasture-based tropical conditions, particularly when nutritional resources are limited. From an economic perspective, even a small deterioration in CI reduces annual calf output and prolongs the period during which cows consume resources without producing saleable offspring. Thus, scenario 2 illustrates that selection emphasis on intermediate-age growth may improve sale weight while simultaneously generating a small but unfavorable correlated response in reproductive efficiency.

Therefore, selection for W16 should be accompanied by explicit emphasis on CI, or by restricted-index strategies, to prevent low reproductive performance. This is particularly important in beef tropical systems, where reproductive efficiency determines calf output and land-use efficiency. Although low-heritability traits such as CI and difficult to measure traits such as LONG were historically underrepresented in selection indices, their inclusion is essential to improve cow fertility, productive permanence, and long-term economic efficiency (Cole et al. 2021).

Although this study considered only traits routinely measured by farmers, continuous monitoring of genetic gains should be complemented with periodic evaluations of production systems, market conditions, and productivity levels (Cole et al. 2021; Souza et al. 2022). Because the estimated genetic gains and economic contributions across the three production scenarios are directly influenced by the genetic and phenotypic covariance structure of the evaluated traits, future updates will be necessary, particularly if additional traits related to production, health, fertility, adaptation, or product quality are incorporated into the selection index (Cole et al. 2021).

Overall, the economic values derived in this study confirm that growth, reproductive efficiency, and longevity traits affect the profitability of Romosinuano cattle production systems in Colombia. Under the evaluated market and production conditions, CI, W16, and AFC emerged as relevant traits for selection because they represent complementary components of system efficiency: reproductive output per cow, biologically efficient post-weaning growth, and replacement female productivity. The consistent economic contribution of LONG further supports the inclusion of adaptive and functional traits in breeding objectives for Romosinuano cattle, particularly because longevity reflects productive permanence under tropical conditions and reduces replacement pressure. Therefore, selection indices for this population should balance growth, fertility, and adaptation-related traits rather than prioritize body weight alone, contributing to more profitable, resilient, and sustainable beef production systems.

## Data Availability

The databases used in this study are not publicly available due to AGROSAVIA’s institutional policies and the confidentiality of the Romosinuano cattle breeders’ data. The procedures described in the R project script are available from the corresponding author on reasonable request.

## References

[CR1] Albera A, Carnier P, Groen AF (2004) Definition of a breeding goal for the Piemontese breed: economic and biological values and their sensitivity to production circumstances. Livest Sci 89(1):66–77. 10.1016/j.livprodsci.2003.12.004

[CR2] Amaya A, Garrick D, Martínez R, Cerón-Muñoz M (2020) Economic values for index improvement of dual-purpose Simmental cattle. Livest Sci 240:104224. 10.1016/j.livsci.2020.104224

[CR3] Amaya A, López PL, Ramírez J (2022) Selection indexes to optimise genetic and economic progress in Colombian Blanco Orejinegro cattle. Livest Sci 263:105015. 10.1016/j.livsci.2022.105015

[CR4] Brumatti RC, Ferraz JBS, Eler JP, Formigoni IB (2011) Development of selection indices in beef cattle using a bioeconomic model. Arch Zootec 60(229):205–213

[CR5] Cañas–Álvarez JJ, Ossa-Saraz GA, Garcés-Blanquiceth JL, Burgos–Paz WO (2023) Genealogical structure of the Colombian Romosinuano Creole cattle. Trop Anim Health Prod 55(5):292 10.1007/s11250-023-03694-1 PMC10435628 37589774

[CR6] Cole JB, Dürr JW, Nicolazzi EL (2021) Invited review: The future of selection decisions and breeding programs: What are we breeding for, and who decides? J Dairy Sci 104:5111–5124. 10.3168/jds.2020-19777 33714581

[CR7] Cushman RA, Kill LK, Funston RN, Mousel EM, Perry GA (2013) Heifer calving date positively influences calf weaning weights through six parturitions. J Anim Sci 91:4486–4491. 10.2527/jas.2013-6465 23825337

[CR8] Damiran D, Larson KA, Pearce LT, Erickson NE, Lardner BHA (2018) Effect of calving period on beef cow longevity and lifetime productivity in western Canada. Transl Anim Sci 2(1):S61–S65. 10.1093/tas/txy020 PMC7200576 32704738

[CR9] Eler JP, Bignardi AB, Ferraz JBS, Santana ML (2014) Genetic relationships among traits related to reproduction and growth of Nelore females. Theriogenology 82(5):708–714. 10.1016/j.theriogenology.2014.06.001 25023297

[CR10] Galeano RAP (2010) Evaluación genética del recurso animal de los sistemas de producción de bovinos en doble propósito en Colombia. Universidad Nacional de Colombia, Bogotá, Colombia. MSc thesis

[CR11] Garrick DJ (2002) Accounting for feed costs in improvement programmes for grazed dairy cattle. In: Proceedings of the 7th world congress on genetics applied to livestock production, Montpellier, France

[CR12] Gathura DM, Muasya TK, Kahi AK (2020) Meta-analysis of genetic parameters for traits of economic importance for beef cattle in the tropics. Livest Sci 242:104306. 10.1016/j.livsci.2020.104306

[CR13] Hazel LN, Lush JL (1942) The efficiency of three methods of selection. J Heredity 33(11):393–399. 10.1093/oxfordjournals.jhered.a105102

[CR14] Hunde D, Tadesse Y, Tadesse M et al (2024) Economic values of some important traits for smallholder dairy production in Central Ethiopia. Trop Anim Health Prod 56:267. 10.1007/s11250-024-04159-9 39305331

[CR15] Kluska S, Ferreira OB, Bonamy M, Justino CH, Braga FF et al (2018) Estimates of genetic parameters for growth, reproductive, and carcass traits in Nelore cattle using the single step genomic BLUP procedure. Livest Sci 216:203–209. 10.1016/j.livsci.2018.08.015

[CR16] Kodak O, Nagy I (2019) Historical overview of the selection indices applied in pig breeding. Acta Agrar Kaposváriensis 23(1):22–31. 10.31914/aak.2294

[CR17] Koots KR, Gibson JP (1998) Economic values for beef production traits from a herd level bioeconomic model. Can J Anim Sci 78(1):29–45. 10.4141/A97-038

[CR18] Kosińska-Selbi B, Schmidtmann C, Ettema JF, Szyda J, Kargo M (2022) Breeding goals for conservation and active Polish dairy cattle breeds derived with a bio-economic model. Livest Sci 255:104809. 10.1016/j.livsci.2021.104809

[CR19] Krupová Z, Krupa E, Zavadilová L, Kašná E, Žáková E (2020) Current challenges for trait economic values in animal breeding. Czech J Anim Sci 65:454–462. 10.17221/161/2020-CJAS

[CR20] Laske CH, Machado TBB, Laurino DNJL, Fernando FCF (2012) Breeding objectives and economic values for traits of low input family-based beef cattle production system in the State of Rio Grande do Sul. R Bras Zootec 41(2):298–305. https://ainfo.cnptia.embrapa.br/digital/bitstream/item/57524/1/a10v41n2.pdf

[CR21] Mancin E, Mantovani R, Tuliozi B, Sartori C (2022) Economic weights for restriction of selection index as optimal strategy for combining multiple traits. J Dairy Sci 105(12):9751–9762. 10.3168/jds.2022-22085 36307238

[CR22] Mezgebe G, Gizaw S, Urge M (2018) Economic values of Begait cattle breeding-objective traits under low and medium input production systems in northern Ethiopia. LRRD 30:12. http://www.lrrd.org/lrrd30/1/gebret30012.html

[CR24] Michaličková M, Krupová Z, Krupa E, Zavadilová L (2016) Economic weights as a tool for sustainable livestock farming. Dissertation. 10.15414/isd2016.s2.05

[CR23] Moorey SE, Biase FH (2020) Beef heifer fertility: importance of management practices and technological advancements. J Anim Sci Biotechnol 11:97. 10.1186/s40104-020-00503-9 PMC7528292 33014361

[CR25] National Research Council - NRC (2021) Nutrient Requirements of Dairy Cattle. Eighth Revised Edition, Washington 38386771

[CR26] Ossa SGA, Suárez MA, Pérez JE (2007) Factores ambientales y genéticos que influyen la edad al primer parto y el intervalo entre partos en hembras de la raza criolla Romosinuano. Ciencia y Tecnología Agropecuaria 8(2):74–80. https://www.redalyc.org/articulo.oa?id=449945023010

[CR27] Ossa G, Narváez H, Noriega J, Abuabara Y, Vergara O (2013) Heredabilidad y tendencias genéticas para características de crecimiento en ganado criollo Romosinuano. ALPA 21(1):394–396. 10.53588/alpa.293407

[CR28] Ossa SGA, López MJL, Quijano BJH, Santana RMO, Garcés BJL (2021) Análisis retrospectivo de caracteres reproductivos en hembras bovinas criollas colombianas Romosinuano. Ciencia y Tecnología Agropecuaria 22(1):e1804. 10.21930/rcta.vol22_num1_art:1804

[CR29] Pérez GJ (2004) Los ciclos ganaderos en Colombia. Documentos de trabajo sobre economía regional No. 46. Banco de la República, Centro de estudios económicos regionales (CEER)- Cartagena, Colombia

[CR30] Pravia MI, Ravagnolo O, Urioste JI, Garrick DJ (2014) Identification of breeding objectives using a bioeconomic model for a beef cattle production system in Uruguay. Livest Sci 160:21–28. 10.1016/j.livsci.2013.12.006

[CR31] Ramírez TE, Ocampo GR, Burgos PW, Elzo MA, Martínez SR, Cerón-Muñoz M (2019) Estimación poligénica y genómico-poligénica para características de crecimiento en ganado Blanco Orejinegro (BON). Livest Res Rural Dev. http://www.lrrd.org/lrrd31/3/ceron31030.html

[CR32] Ramírez TE, Burgos PW, Elzo MA, Martínez SR, Cerón-Muñoz M (2020) Genetic parameters and trends for growth traits in Blanco Orejinegro cattle. Transl Anim Sci 4(3):txaa174. 10.1093/tas/txaa174 PMC7592852 33134877

[CR33] Ratwan P, Chakravarty AK, Kumar M (2021) Development of a sustainable performance index based on production, reproduction, health and longevity for genetic improvement of Sahiwal cattle. Reprod Domest Anim 56(5):792–800. 10.1111/rda.13919 33624363

[CR34] Restrepo JAN (2014) Estimación de parámetros genéticos para la stayability y su asociación con otras características de interés económico en la raza colombiana Lucerna. MSc thesis. Universidad Tecnológica de Pereira, Pereira, Colombia

[CR35] Samaraweera AM, van der Werf JHJ, Boerner V, Hermesch S (2022) Economic Values for Production, Fertility and Mastitis Traits for Temperate Dairy Cattle Breeds in Tropical Sri Lanka. J Anim Breed Genet 139:330–341. 10.1111/jbg.12667 PMC9306856 35072970

[CR36] Schmidtmann C, Thaller G, Kargo M, Hinrichs D, Ettema J (2021) Derivation of economical values for German dairy breeds by means of a bio-economic model-with special emphasis on functional traits. J Dairy Sci 104(3):3144–3157. 10.3168/jds.2019-17635 33358794

[CR37] Schneeberger M, Barwick SA, Crow GH, Hammond K (1992) Economic indexes using breeding values predicted by BLUP. J Anim Breed Genet 109:180–187. 10.1111/j.1439-0388.1992.tb00395.x

[CR38] Shi R, van der Linden A, Oosting S, Wang Y, Ducro B (2025) Derivation of Economic Values for Breeding Objective Traits of Chinese Holstein Dairy Cows Using a Bio-Economic Model. J Anim Breed Genet 142:513–527. 10.1111/jbg.12922 PMC12340353 39775954

[CR39] Souza FM, Lopes FB, Rosa GJM, Fernandes R, Magnabosco VS, Magnabosco CU (2022) Genetic selection of Nellore cattle raised in tropical areas: economic indexes and breeding decisions risks. Livest Sci 265:105098. 10.1016/j.livsci.2022.105098

[CR40] Wahinya PK, Jeyaruban MG, Swan AA, van der Werf JHJ (2022) Breeding objectives for dairy cattle under low, medium and high production systems in the tropics. Animal 16(5):100513. 10.1016/j.animal.2022.100513 35436647

[CR41] Wolfová M, Wolf J, Přibyl J, Zahrádková R, Kica J (2005) Breeding objectives for beef cattle used in different production systems: 1. Model development. Livest Prod Sci 95(3):201–215. 10.1016/j.livprodsci.2004.12.018

[CR42] Zhang XL, Amer P (2021) A new selection index percent emphasis method using subindex weights and genetic evaluation accuracy. J Dairy Sci 104:5827–5842. 10.3168/jds.2020-19547 33663843

